# Effects of culinary treatments on the lipid nutritional quality of fish and shellfish

**DOI:** 10.1016/j.fochx.2023.100856

**Published:** 2023-09-04

**Authors:** Karsoon Tan, Leiheng Huang, Kianann Tan, Leongseng Lim, Ya Peng, Kit-Leong Cheong

**Affiliations:** a College of Marine Science, Guangxi Key Laboratory of Beibu Gulf Biodiversity Conservation, Beibu Gulf Ocean Development Research Centre, Beibu Gulf University, Qinzhou, Guangxi, China; b Guangdong Provincial Key Laboratory of Aquatic Product Processing and Safety, College of Food Science and Technology, Guangdong Ocean University, Zhanjiang 524088, China; c Borneo Marine Research Institute, Universiti Malaysia Sabah, Kota Kinabalu, Sabah, Malaysia

**Keywords:** Bivalves, Culinary treatments, Lipid nutritional quality, Coronary heart disease, Prevention

## Abstract

Coronary heart disease (CHD) is one of the leading causes of death worldwide. Seafood, especially fish and shellfish, is a healthy food that reduces the risk of CHD. In many regions, seafood is consumed cooked to eliminate potentially pathogenic microorganisms. Although there have been many reports of culinary preparations causing changes in the fatty acid profile of fish and shellfish, this information has not been well organized, and most of it is not associated to CHD. Therefore, this study was conducted to study the effect of culinary treatments of seafood on lipid nutritional quality in relation to promotion/prevention of CHD. In this study, fatty acid profiles of fish and shellfish prepared with different culinary preparations were obtained from published literature. Lipid nutritional quality indices related to promoting/preventing CHD were calculated and analyzed to reveal the effects of culinary treatment on the lipid nutritional quality of fish and shellfish in promoting/preventing of CHD. The information in this article is very useful and can fill the knowledge gap of the effects of culinary preparation on the lipid nutritional quality of fish and shellfish. Such information is very useful for guiding consumers to choose better ways to cook fish and shellfish to reduce the risk of CHD.

## Highlights

• High temperature and long heat treatment generally impairs EPA + DHA, n-3/n-6.
• High temperature and long heat treatment cause hydrolysis of pro-atherogenic SFA.
• Effects of frying on lipid nutritional quality depend on frying medium used.
• The worse culinary treatment for fish and shellfish is frying using margarine.

## Introduction

Coronary heart disease (CHD), also known as coronary artery disease, is a phenomenon where cholesterol deposits on the artery wall, causing the coronary arteries to become too narrow and unable to obtain the optimum oxygen supply. Since 1970, CHD has been the leading cause of global death and disability (Arroyo-Quiroz et al., 2020). Although the mortality rate of CHD in developed countries has continued to decline due to advances in prevention and treatment since the 1970s, it remains the largest contributor to mortality in most developed countries (GBD, 2018). In fact, the mortality of CHD has increased in most developing and undeveloped countries (Arroyo-Quiroz et al., 2020). To date, CHD is the third leading cause of mortality worldwide, with 17.8 million deaths annually (Brown et al., 2022). Early epidemiological studies found that Alaskan Natives and Greenland Eskimos who ate large amounts of seafood, had a lower mortality rate of CHD (Kromann and Green, 1980, Newman et al., 1993). Since then, many studies have been conducted to investigate the relationship between seafood intake and the incidence rate of CHD. Although some findings do not show a clear relationship between seafood consumption and the incidence rate of CHD (e.g. Bechthold et al., 2017; Hengeveld et al., 2018), many evidence have shown that high consumption of seafood contributes to the prevention of CHD (Kromhout et al., 2002; Zheng et al., 2012; He et al., 2004; Whelton et al., 2004, Zhang et al., 2020).

Seafood, especially fish and shellfish, is high quality animal protein that rich in polysaccharides (e.g. Tan et al., 2023a), carotenoids (e.g. Tan et al., 2022a) and omega-3 long-chain polyunsaturated fatty acids (n-3 LC-PUFA), especially eicosapentaenoic acid (EPA; C20:5n-3) and docosahexaenoic acid (DHA; C22:6n-3), have well-established beneficial properties for human health (Ajith and Jayakumar, 2018, Tan et al., 2021). Dietary guidelines recommend consuming two servings of seafood per week to prevent CHD (e.g. Arnett et al., 2019, Mohan et al., 2021). However, very low fish intake (<1 per week) is common in many regions, including America, Europe, Middle East, Southeast Asia and Africa (Stark et al., 2016, Hengeveld et al., 2018). In fact, a global dietary n-3 LC-PUFA survey based on EPA + DHA intake data revealed that>80% of the global population consumes<250 mg of n-3 LC-PUFA per day (minimum recommended value), while the global average intake of n-3 LC-PUFA is only 163 mg/day (Micha et al., 2014). Based on a *meta*-analysis, Stark et al. (2016) revealed that populations with access to adequate seafood (EPA + DHA in blood of > 8%) were limited to a few regions, including the Sea of Japan, Scandinavia, and areas with indigenous populations.

Culinary preparation is an important process for eliminating pathogenic microorganisms in food (e.g. Lee et al., 2008). However, at the same time, culinary treatment may lead to a decrease in the lipid nutritional quality of seafood, mainly due to heat-induced oxidation of n-3 PUFAs and the addition of exogenous oils that can modify the original fatty acid profile (e.g. Sampels, 2015, Biandolino et al., 2021). In addition, due to the limited accessibility for the world population in many regions to achieve optimal levels of fish and shellfish (Tan et al., 2020), monitoring the lipid nutritional quality of fish and shellfish during culinary preparation can serve as an alternative to monitoring the lipid nutritional quality of fish and shellfish for maximum CHD prevention effect. To date, most studies evaluating seafood consumption and CHD have focused on its relationship with the risk of CHD, and it is recommended to consume it once a week (e.g. Yang et al., 2016, Jayedi et al., 2018, Zhao et al., 2019, Zhang et al., 2020). However, the effects of culinary preparation of seafood on lipid nutritional quality is still unclear.

In this study, lipid profiles of fish and shellfish prepared with different culinary treatments were retrieved from the published literature to calculate various lipid nutritional quality indicators related to promoting or preventing CHD, including EPA + DHA, n-3:n-6, (MUFA + PUFA)/SFA-C18:0, atherogenic index (AI), thrombogenic index (TI) and hypocholesterolemic/hypercholesterolemic index (H/H). The changes (%) of these indicators in fish and shellfish prepared with different culinary preparations (relative to raw fish or shellfish) were then calculated and analyzed. To the best of our knowledge, this article represents the first comprehensive analysis of the effects of culinary treatments on the lipid nutritional quality of fish and shellfish in relation to the risk of CHD. The information in this article is very useful for understanding the effects of culinary preparation on the lipid nutritional quality of fish and shellfish. Such information will aid to provide guidance to consumers in choosing better culinary preparations to maximize the lipid nutritional quality of fish and shellfish, and to maximize the effectiveness of preventing CHD.

## Materials and methods

### Literature search and data collection

Articles were obtained from Web of Science and Google Scholar (up to July 2022) using keywords such as “fatty acid profile of fish”, “fatty acid profile of shellfish”, “culinary preparation” and/or “cooking”. In order to obtain any articles that may be missed in the online search, relevant articles in the reference list of each article were downloaded. This procedure was repeated until no new articles were found. As a result, a total of 3678 articles were obtained from the literature search.

All articles (n = 3678) were further screened for complete fatty acid profile table of raw and cooked fish or shellfish. After screening, only 76 articles met the requirements, and this comprehensive literature search and screening provided us with a reasonable number of fatty acid profiles of fish (n = 235) and shellfish (n = 69) to examine the effects of culinary treatment on the lipid nutritional quality of fish and shellfish in relation to the risk of CHD.

In order to verify the effect of culinary preparations on fatty acid content, the results were calculated on a dry weight basis. Moreover, in order to enable a comparison of the results from different studies, all data were expressed as a percentage of fatty acids to total fatty acids.

### Data extraction and statistical analysis

From each fatty acid profile, we collected general information of the common name and scientific name of each analyzed species, the culinary treatment used, and the types of oil used if frying is involved. We also calculated various lipid nutritional quality indicators associated with prevention/promotion of CHD, including EPA + DHA (%), n-3:n-6, PUFA/SFA, (MUFA + PUFA)/SFA-C18:0, atherogenic index (AI), thrombogenic index (TI) and hypocholesterolemic/hypercholesterolemic index (H/H).

Omega-3 long-chain unsaturated fatty acids (n-3 LC-PUFAs), especially highly unsaturated ones (EPA and DHA) display several properties such as antithrombotic, antiinflammatory, antiarrhythmic and vasodilatory (Lombardo and Chicco, 2006). In addition, it is generally believed that consumption of high proportions of pro-inflammatory n-6 PUFA can lead to various adverse health effects (e.g. higher risk of cancer, ulcerative colitis), and equilibrate ratio of n-3 PUFA and n-6 PUFA can help in the prevention and treatment of many diseases (e.g hypertension and coronary artery problems) (Kinsella et al., 1990). Therefore, food rich in EPA and DHA with an n-3: n-6 ratio of > 0.45 (preferably > 1) are important indicators of healthy food to prevent CHD (Bhardwaj et al., 2016).

Saturated fatty acids (SFA) consumption, especially palmitic acid (C16:0) and myristic acid (C14:0), is associated with CHD by increasing low-density lipoprotein (LDL) cholesterol. As a result, since 1970, a reduction in SFA consumption has been recommended to reduce the risk of CHD (Mozaffarian et al., 2010). Therefore, PUFA/SFA has become an important indicator for assessing the risk of CHD and lipid nutritional quality of food. According to Health and Social Security, the recommended minimum value for the PUFA/SFA ratio is > 0.45, and ratios below this value are detrimental to human health (HMSO, 1994). Recently, many studies have shown that a diet rich in monounsaturated fatty acids (MUFA) has many health benefits, especially decreasing the risk factors of CHS (total cholesterol, LDL cholesterol and triglycerides) and increasing the high density lipoprotein cholesterol (Mozaffarian et al., 2010, Siri-Tarino et al., 2010). Considering the health benefits of MUFA and the fact that some SFAs (especially stearic acid (C18:0)) do not increase the risk of CHD, (MUFA + PUFA)/(SFA-C18:0) is another important lipid nutritional quality indicators assess the risk of CHD.

The atherogenic (AI) and thrombogenic indices (TI) were proposed by Ulbricht and Southgate (1991) as indicators of the stimulus potential of platelet aggregation, of which is used to evaluate the potential of food to influence the incidence of CHD. In particular, the low values of AI and TI suggest the higher levels of anti-atherogenic and anti-thrombogenic fatty acids and the greater potential to prevent the onset of CHD (Weber et al., 2008). As for the hypocholesterolemic/hypercholesterolemic (H/H) fatty acid ratio, the specific effects of fatty acids on the metabolism of lipoproteins transporting plasma cholesterol were considered, where lower values of HH are considered detrimental to human health. Therefore, a high HH value is desirable food with health benefits. AI, TI and H/H were calculated according to equations (1), (2) and (3), respectively (Ulbricht and Southgate, 1991, Santos-Silva et al., 2002):

(1) AI = (12:0 + (4 *×* 14:0) + 16:0)/(∑MUFA + ∑PUFA (*n*-6) + (*n*-3))

(2) TI = (14:0 + 16:0 + 18:0)/((0.5 *×* ∑MUFA) + (0.5 *×* ∑PUFA (*n*-6)) + (3 *×* ∑PUFA (*n*-3)) + (*n*-3)/(*n*-6))

(3) H/H = (18:1*n*-9 + 18:2*n*-6 + 20:4*n*-6 + 18:3*n*-3 + 20:5*n*-3 + 22:5*n*-3 + 22:6*n*-3)/(14:0 + 16:0)

The changes (%) in these indicators in fish and shellfish prepared with different culinary treatments (relative to raw fish and shellfish) were then calculated and analyzed. Prior to analysis, all data was translated (X + 1-min(X)) to ensure that the smallest number is 1, and then log_10_ transformed. All statistical analyses were done on SPSS Windows Statistical Package TM (version 21), and significance for all analyses was set to *P* < 0.05 unless noted otherwise. Prior to analyses, all variables were tested for normality and variance homogeneity by Kolmogorov–Smirnov and Levene's tests, respectively. One-way ANOVA was performed followed by Turkey multiple comparison tests (Turkey HSD) to test for significant differences in EPA + DHA, n-3/n-6, PUFA/SFA, (MUFA + PUFA)/(SFA-C18:0), AI, TI and H/H among raw and cooked fish and shellfish.

## Results

### Effects of culinary treatments on the EPA + DHA and n-3/n-6 ratio of fish and shellfish

**Fig. 1:**
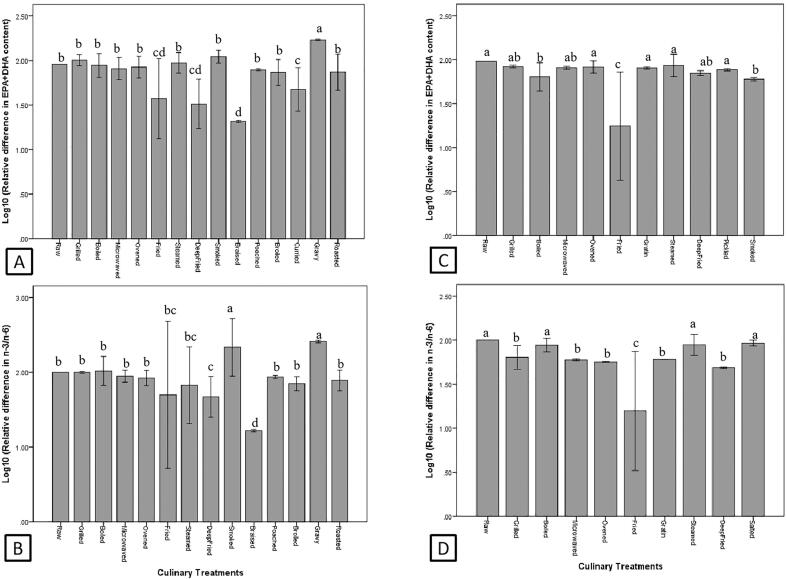
Effects of culinary treatments on EPA + DHA and n-3/n-6 of fish (A and B) and shellfish (C and D). Different letters indicate significant difference (P < 0.05).

**Fig. 2:**
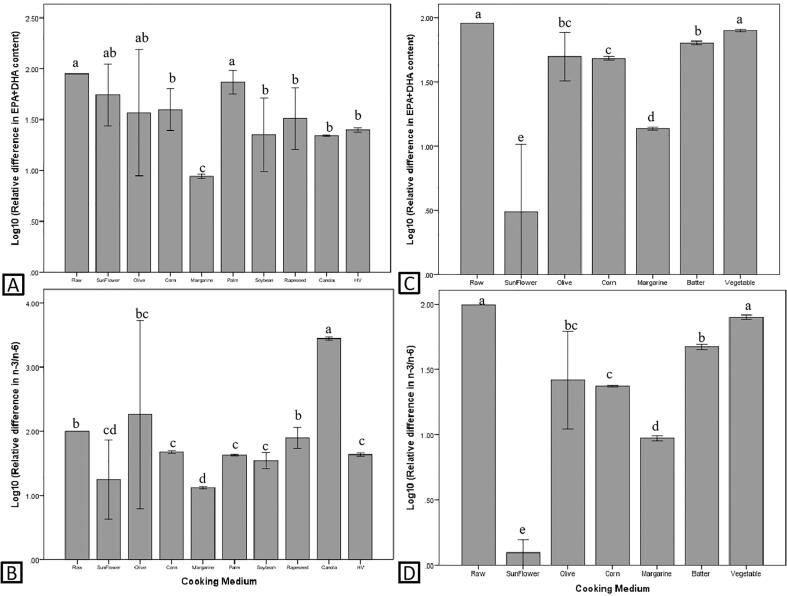
Effects of cooking mediums on EPA + DHA and n-3/n-6 of fried fish (A and B) and shellfish (C and D). HV = hydrogenated vegetable oil and different letters indicate significant difference (P < 0.05).

**Fig. 3:**
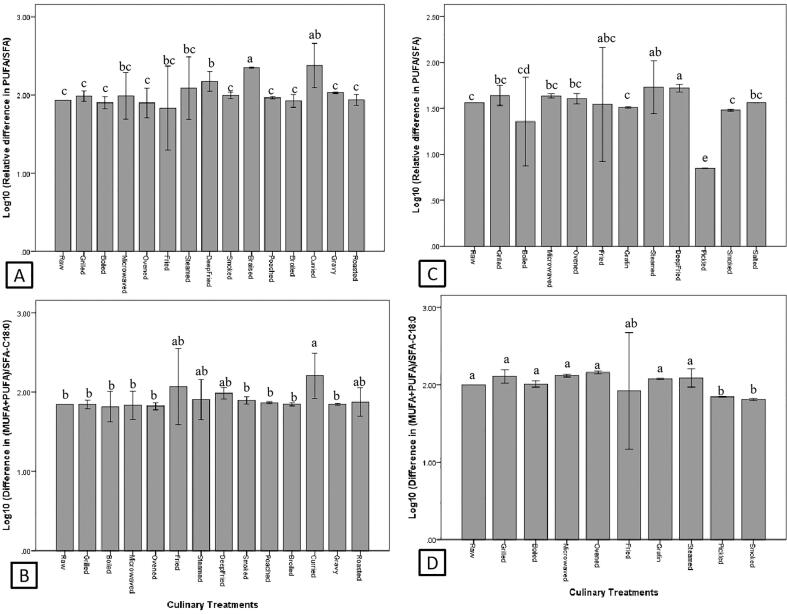
Effects of culinary treatments on PUFA/SFA and (MUFA + PUFA)/SFA-C18:0 of fish (A and B) and shellfish (C and D). Different letters indicate significant difference (P < 0.05).

The effects of culinary treatment on EPA + DHA and n-3/n-6 of fish and shellfish are illustrated in Fig. 1. According to the study on the influence of culinary treatments on the EPA + DHA content (n = 177) and n-3/n-6 (n = 134) of fish, some culinary treatments (especially frying and braising) significantly impaired (*P* < 0.05) the EPA + DHA (Fig. 1A) and n-3/n-6 (Fig. 1B) of fish. The reduction of EPA + DHA and n-3/n-6 highly depends on the oil used (Fig. 2), among which margarine caused the greatest reduction (*P* < 0.05) in both EPA + DHA and n-3/n-6. In addition, frying fish with corn oil, soybean oil, rapeseed oil, canola oil and hydrogenated vegetable oil also significantly reduced (*P* < 0.05) the EPA + DHA content of fried fish (Fig. 2A). As for n-3/n-6, frying fish with sunflower oil, corn oil, palm oil, soybean oil and hydrogenated vegetable oil also cause significant reduction (*P* < 0.05) in n-3/n-6, but frying fish with canola oil caused significant increment (*P* < 0.05) in n-3/n-6 (Fig. 2B).

As for shellfish (n = 40), frying had the greatest negative impact (*P* < 0.05) on EPA + DHA and n-3/n-6. In addition, boiling and smoking also significantly reduced the EPA + DHA content of shellfish (Fig. 1C), while grilling, microwave cooking, oven cooking, grating and deep frying caused significant reduction (*P* < 0.05) in n-3/n-6 of shellfish (Fig. 1D). Similar to fish, the decreased of EPA + DHA and n-3/n-6 in fried shellfish is highly depends on the frying medium used, in which frying with sunflower oil caused the greatest reduction (*P* < 0.05) in EPA + DHA and n-3/n-6, followed by margarine, corn oil, olive oil and batter (*P* < 0.05) (Fig. 2C and 2D).

### Effects of culinary treatments on the PUFA/SFA and (MUFA + PUFA)/SFA-C18:0 of fish and shellfish

The effects of culinary treatment on PUFA/SFA (n = 175) and (MUFA + PUFA)/SFA-C18:0 (n = 143) are summarized in Fig. 3. In general, baking and curry cooking significantly increased (*P* < 0.05) the PUFA/SFA of fish (Fig. 3A), while curry cooking significantly increased (*P* < 0.05) the (MUFA + PUFA)/SFA-C18:0 of fish (Fig. 3B). As for frying, the changes of PUFA/SFA and (MUFA + PUFA)/SFA-C18:0 of fried fish were highly affected by the frying medium, and the negative impact was the greatest (*P* < 0.05) when fried with margarine. However, frying with canola oil, rapeseed oil and soybean oil significantly improved (*P* < 0.05) the PUFA/SFA and (MUFA + PUFA)/SFA-C18:0 of fried fish.

**Fig. 4:**
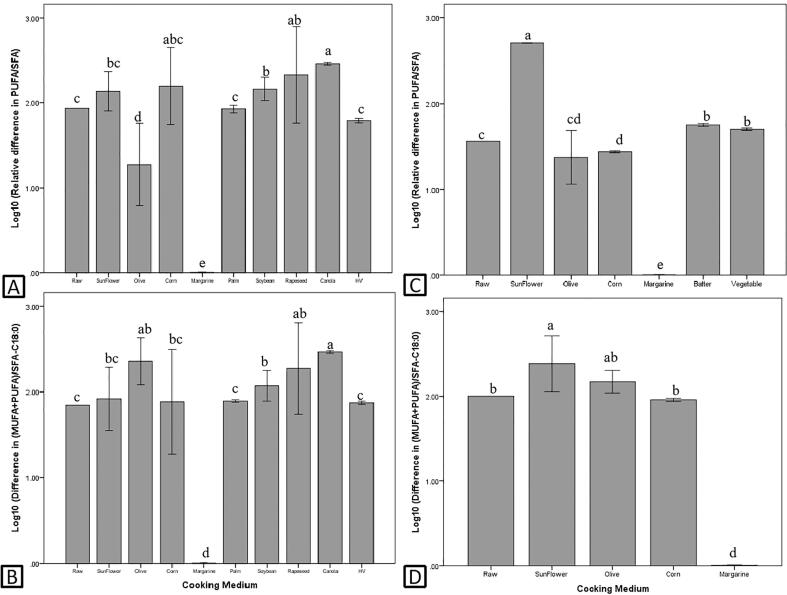
Effects of cooking mediums on PUFA/SFA and (MUFA + PUFA)/SFA-C18:0 of fried fish (A and B) and shellfish (C and D). HV = hydrogenated vegetable oil and different letters indicate significant difference (P < 0.05).

In shellfish, the results of the effects of culinary preparations on PUFA/SFA (n = 50) and (MUFA + PUFA)/SFA-C18:0 (n = 35) revealed that steaming and deep frying significantly increased (*P* < 0.05), while pickling significantly decreased (*P* < 0.05) the PUFA/SFA of shellfish. On the other hand, pickling and smoking significantly decreased the (*P* < 0.05) (MUFA + PUFA)/SFA-C18:0 of shellfish. As for frying, when sunflower seed oil and margarine were used, PUFA/SFA and (MUFA + PUFA)/SFA-C18:0 of shellfish were the highest (*P* < 0.05) (Fig. 4C) and lowest (*P* < 0.05) (Fig. 4D), respectively. In addition, frying shellfish with batter and vegetable oil also significantly increased (*P* < 0.05) the PUFA/SFA.

### Effects of culinary treatments on the atherogenicity, thrombogenicity and hypocholesterolaemic/ hypercholesterolaemic indices of fish and shellfish

**Fig. 5:**
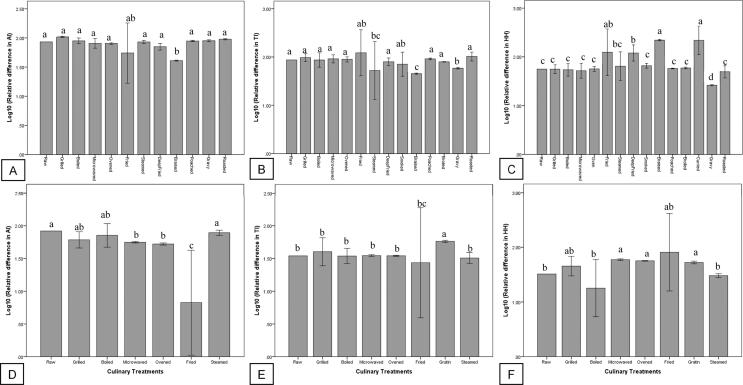
Effects of culinary preparations on the atherogenicity (AI), thrombogenicity (TI) and hypocholesterolaemic/ hypercholesterolaemic (HH) indices of fish (A, B and C) and shellfish (D, E and F). Different letters indicate significant difference (P < 0.05).

**Fig. 6:**
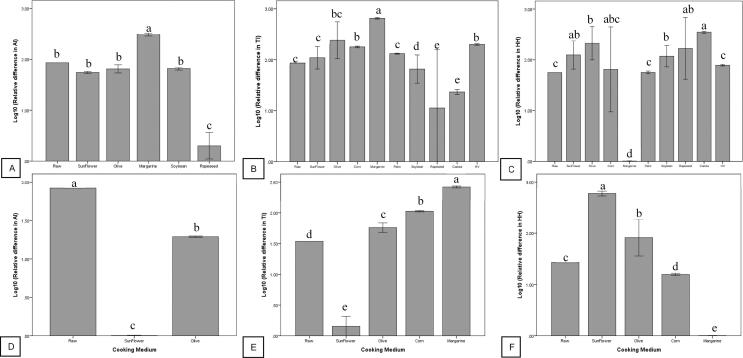
Effects of cooking mediums on the atherogenicity (AI), thrombogenicity (TI) and hypocholesterolaemic/ hypercholesterolaemic (HH) indices of fried fish (A, B and C) and shellfish (D, E and F). HV = hydrogenated vegetable oil and different letters indicate significant difference (P < 0.05).

A total of 37, 99 and 99 studies associated with the effects of culinary treatment on the AI, TI and H/H of fish and shellfish are summarized in Fig. 5. In general, braising led to significant reduction (*P* < 0.05) in AI index, steaming, braising and graving caused significant reduction (*P* < 0.05) in TI index, and graving caused significant reduction (*P* < 0.05) in HH index. However, frying, braising and curry cooking significantly increased (*P* < 0.05) the HH index of fish. As for frying, frying with margarine and rapeseed caused significant increase (*P* < 0.05) and decrease (*P* < 0.05) of AI index, respectively (Fig. 6A). Frying with margarine, corn oil and hydrogenated vegetable oil significantly increased (*P* < 0.05) TI index, but frying with soybean oil, rapeseed oil and canola oil significantly decreased (*P* < 0.05) TI index (Fig. 6B). Frying with olive oil, soybean oil, rapeseed oil and canola oil significantly increased (*P* < 0.05) HH index, but frying with margarine significantly decreased (*P* < 0.05) HH index (Fig. 6C).

In shellfish, a total of 20, 31 and 31 studies addressed the effects of culinary treatments on AI, TI and H/H. In general, frying, microwave cooking and oven cooking significantly decreased (*P* < 0.05) the AI index of shellfish (Fig. 5D), grating significantly increased (*P* < 0.05) the TI index of shellfish (Fig. 5E), and microwave cooking, oven cooking and grating significantly increased (*P* < 0.05) the HH index of shellfish (Fig. 5F). As for frying, frying with sunflower oil and olive oil caused significant reduction (*P* < 0.05) in the AI index of shellfish (Fig. 6D). Frying with sunflower oil caused significant reduction (*P* < 0.05), but frying with olive oil, corn oil and margarine caused significant increment (*P* < 0.05) in the TI index of shellfish (Fig. 6E). Frying with corn oil and margarine caused significant reduction (*P* < 0.05), but frying with sunflower oil and olive oil caused significant increment (*P* < 0.05) in the HH index of shellfish (Fig. 6F).

## Discussion

In general, studies have shown that high temperatures (e.g frying) and long period of heat treatment (e.g braising, roasting, smoking and curry cooking) during culinary preparation caused significant reduction in EPA + DHA and n-3/n-6 levels in fish and shellfish. EPA and DHA are very sensitive to oxidation, mainly due to the high degree of unsaturation (Weber et al., 2008, Tan et al., 2022b, Tan et al., 2023b). Oxidative degradation of LC-PUFA caused production of various radicals, –CHO, –CO and –CH3 that subsequently produce methylglyoxal, 2,3-butanedione, α,β-unsaturated aldehydes, α-dicarbonyl compounds and etc. (Ma et al., 2019). Therefore, it is not surprising that high temperature or prolong heat treatment during culinary preparation will lead to the oxidation of unsaturated fatty acids. Since EPA and DHA are the major fatty acids in fish and shellfish, the reduction in EPA and DHA also caused a significant reduction in the n-3/n-6 ratio. Interestingly, graving significantly increased the EPA + DHA content in fish. For example, the EPA + DHA content of (11.49%) Indian mackerel *Bastrilliger kanagurta* in gravy was significantly higher than that of raw mackerel (6.42%) (Marichamy et al., 2009), which may be associated with condensation of fish tissues and the release of EPA and DHA during cooking (Sampels, 2015).

Among culinary treatments, frying has the greatest negative impact on EPA + DHA and n-3/n-6 in seafood, especially shellfish. This is mainly due to the exchange of fatty acids between food and cooking oils, resulting in a significant decrease in the content of some important fatty acids in fried food, especially EPA and DHA (Sanchez-Muniz et al., 1992, Zotos et al., 2013, Ghribi et al., 2017, Choo et al., 2018, Bejaoui et al., 2019, Biandolino et al., 2021). For example, Zotos et al., (2013) studied the changes of fatty acids in anchovy *Engraulis encrasicholus* and cooking oil (olive oil and sunflower oil) caused by frying. The results revealed that EPA + DHA (from 33.36 to 45.12% to 1.34–3.95%) and n-3/n-6 (from 12.15 to 15.62 to 0.06–2.56) of anchovy decreased significantly, while EPA + DHA (from not detected to 0.21–0.34) and n-3/n-6 (from 0.13 to 22–35.25) of cooking oil increased significantly. Ghribi et al. (2017) compared the fatty acid composition of *Arca noae* prepared with different culinary treatments, including steaming, boiling, grilling and frying (using olive oil). It was found that fried *Arca noae* contained significantly lower EPA + DHA (3.05%) and n-3/n-6 (0.27) than that of steamed, boiled and grilled *Arca noae* samples (EPA + DHA = 7.33–9.03%; n-3/n-6 = 1.38–1.85). Bejaoui et al. (2019) compared the changes in fatty acids of clam *Veneruois decussata* fried with different cooking oils (corn oil, olive oil and margarine oil). The results revealed that frying increased the n-6 PUFA (5.0% to 10.8–14.1%) but decreased n-3 PUFA (from 26.4% to 7.9–17.4%) of clam, while reducing the n-6 PUFA and increasing n-3 PUFA of cooking oils (especially in margarine). Another good example is that Biandolino et al. (2021) compared the fatty acid profiles of blue mussel *Mytilus galloprovincialis* under different culinary treatments (grilling, boiling, microwave cooking, oven cooking and frying with sunflower oil). It was found that the retention of EPA + DHA and n-3/n-6 in fried mussels (EPA + DHA = 4.60%; n-3/n-6 = 0.15) were significantly lower than that of mussels prepared from other cooking methods (EPA + DHA = 18.32–21.11%; n-3/n-6 = 3.23–3.84).

It is worth noting that the reduction of EPA + DHA in fried fish and shellfish depends largely on the type of frying medium used, with the greatest reduction when frying with margarine and sunflower oil for fish and shellfish, respectively. Since different cooking oils contain different fatty acid compositions, it is not surprising that the fatty acid composition of fried fish and shellfish using different cooking oils change differently. This is mainly attributed to the absorption of oil and fatty acids in fish and shellfish during the frying process (Agren and Hamminen, 1993; Echarte et al., 2001, Gladyshev et al., 2006, Sioen et al., 2006, Weber et al., 2008, Bejaoui et al., 2019). For example, Sioen et al. (2006) compared the fatty acid profile of cod *Gadus morhua* submitted to frying using margarine and olive oil. It was found that both frying medium significantly reduce the EPA + DHA (from 49.61% to 9.83–12.27%) and n-3/n-6 (from 10.00 to 1.37 to 2.50) of *Gadus morhua*, with margarine caused significantly greater reduction than that of olive oil. Similarly, Weber et al. (2008) studied the changes in fatty acid profile of silver catfish *Rhamdia quelen* submitted to frying using different cooking oils (soybean oil, canola oil and hydrogenated vegetable oil). The results revealed that compared to *Rhamdia quelen* fried with canola oil, the reduction of EPA + DHA and n-3/n-6 in fish fried with hydrogenated vegetable oil and soybean oil was greater. Bejaoui et al. (2019) investigated the effect of three frying oils (corn, olive and margarine) on the lipid profile of clams (*Venerupis decussata*). The results revealed that frying with margarine caused the highest reduction in EPA + DHA (from 17.41 to 4.06%) and n-3/n-6 (from 5.30 to 0.56), followed by frying with corn oil (EPA + DHA = 10.27%; n-3/n-6 = 1.29) and olive oil (EPA + DHA = 12.02%; n-3/n-6 = 1.62). These observations indicate that frying fish and shellfish with cooking oils low in unsaturated fatty acids (e.g. margarine) results in significantly higher reduction of EPA + DHA and n-3/n-6 than using cooking oils rich in unsaturated fatty acids (e.g. olive oil). It is worth noting that although the culinary treatments reduced the n-3:n-6 ratio, except for some fried fish (Candela et al., 1998, Hosseini et al., 2014) and shellfish samples (Ghribi et al., 2017, Bejaoui et al., 2019, Biandolino et al., 2021), the n-3/n-6 of most cooked marine fish and marine shellfish is still higher than the recommended standard, indicating that marine fish and shellfish are still good for consumption after culinary preparation.

Some culinary treatments significantly increased PUFA/SFA and (MUFA + PUFA)/SFA-C18:0 of fish (e.g braising and curry cooking) and shellfish (e.g deep frying). For example, deep frying Australian Bass Strait scallop, *Pecten fumatus*, in batter increased PUFA/SFA from 1.31 to 1.56 (Su and Babb, 2007). The curry cooking of Narrow-barred Spanish mackrel, *Scomberomorus commerson*, increased PUFA/SFA and (MUFA + PUFA)/SFA-C18:0 by 1.45 and 1.19 fold, respectively, which was due to the significantly increased in 18:3n-3 and 18:3n-6 (Musaiger and D’Souza, 2011). Similarly, braising of *Cynoscion leiarchus* increased PUFA/SFA by 2.36 fold, due to a significantly higher increased in PUFA (especially 18:3n-3 and 18:3n-6) compared to SFA (Brito et al., 2019). As for frying, similar to EPA + DHA and n-3/n-6, the PUFA/SFA and (MUFA + PUFA)/SFA-C18:0 of fish and shellfish fried in margarine were much lower than the control. This observation further indicates that frying fish and shellfish with a frying medium rich in saturated fats is less healthy.

In general, none of the culinary treatment can significantly increase the AI index of fish and shellfish, indicating that cooking does not increase the risk of CHD. In fact, some culinary treatments decrease the AI (improve health benefits) of fish and shellfish. This is mainly attributed to high level of heat induced hydrolysis of pro-atherogenic SFA (C12:0, C14:0 and C16:0) and an increase of MUFA (e.g. Marichamy et al., 2009, Domiszewski et al., 2011, Merdzhanova et al., 2018, Brito et al., 2019, Biandolino et al., 2021). As for frying, frying with margarine and rapeseed oil caused a significant increase and decrease in AI in fried fish, mainly due to the high and low saturation of the frying medium, respectively (Türkkan et al., 2008, Marichamy et al., 2009, Alexi et al., 2019). For bivalves, frying with sunflower oil and olive oil significantly reduced AI index. Caution through, the AI index of most studies cannot be calculated because the content or composition of C12:0 was not provided. Therefore, more data are needed to further confirm the effects of frying medium on AI of seafood.

Similar to AI, some culinary treatments (e.g. steaming, braising and graving) reduce the TI of fish, indicating that these culinary treatments are more healthy through increasing the content of anti-thrombogenic fatty acids (e.g. de Castro et al., 2007, Marichamy et al., 2009, Koubaa et al., 2012, Brito et al., 2019). In terms of frying, most cooking oils can increase the TI of fish and shellfish, while frying with soybean oil, rapeseed oil and canola oil reduces the TI of fish, as well as frying with sunflower oil reduced the TI of shellfish. This is due to the fact that the absorption rate of cooking oil by food is highly dependent on the viscosity of cooking oil (Yang et al., 2020), of which the viscosity of soybean oil and canola oil is lower than other vegetable oils (Sahasrabudhe et al., 2017). In fact, Zotos et al. (2013) have shown that sunflower oil can penetrate into the flesh of anchovy *Engraulis encrasicholus* even within 3 min of frying. Therefore, the high absorption of cooking oils (rich in n-6) consequent in lower n-3/n-6 ratio, which helps to reduce TI in cooked seafood. As regards to H/H, many culinary treatments can improve the H/H of fish (frying, braising and curry cooking) and shellfish (microwave cooking, oven cooking and grating), indicating a lower risk of cardiovascular diseases. The increased in H/H is mainly attributed to the decrease in C14:0 and C16:0 (e.g. Hosseini et al., 2014, Ghribi et al., 2017, Alexi et al., 2019, Felici et al., 2019, Brito et al., 2019, Bejaoui et al., 2019, Golgolipour et al., 2019, Biandolino et al., 2021).

Taken together, braising, curry cooking, graving, canola oil frying, rapeseed oil frying and soybean oil frying are recommended culinary treatments for fish (Table 1). For shellfish, microwave cooking, oven cooking, olive oil frying and sunflower oil frying are recommended. It is worth noting that although most recommended culinary treatments caused a reduction of EPA + DHA and n-3/n-6, marine fish and shellfish are rich in EPA + DHA and have a high n-3/n-6 ratio, in which cooked fish and shellfish still maintain a high levels of EPA + DHA and the n-3/n-6 ratio higher than the recommended value of > 0.45 (Bhardwaj et al., 2016). Since different culinary treatments have their own advantages and disadvantages, consumers can choose culinary treatments based on their personal interest in specific fatty acids/ indices. On the other hand, the worse way to cook fish and shellfish is frying using margarine. This is due to the fact that it results in the greatest reduction in EPA + DHA, n-3/n-6, PUFA/SFA, (MUFA + PUFA)/SFA-C18:0, AI, TI and H/H of fish and shellfish, indicating an increased risk of CHD. In addition, it is also not recommended to fry shellfish with corn oil, as this will increase the pro-thrombogenic saturated fatty acids and cause a major negative impact on the lipid nutritional quality of shellfish (Table 2).

**Table 1:** Effects of culinary treatments on the lipid nutritional quality of fish.

Common name	Species	Cooking methods	Total lipid (% DW)	EPA + DHA (%)	n3/n6	PUFA/SFA	(MUFA + PUFA)/(SFA - C18:0)	AI	TI	H/H	References
seabream	*Sparus aurata*	Steamed, oven cooked and fried (olive oil)	Fresh = 22.69; steamed = 24.69; oven cooked = 28.81; fried = 27.06	Fresh = 7.76; steamed = 7.54; oven cooked = 7.90; fried = 6.41	Fresh = 0.71; steamed = 0.69; oven cooked = 0.70; fried = 0.69	Fresh = 0.97; steamed = 0.97; oven cooked = 0.99; fried = 0.90	–	Fresh = 0.41; steamed = 0.41; oven cooked = 0.40; fried = 0.33	Fresh = 0.39; steamed = 0.39; oven cooked = 0.38; fried = 0.36	Fresh = 2.95; steamed = 2.95; oven cooked = 3.04; fried = 3.80	Alexi et al., 2019
meagre	*Argyrosomus regius*	Steamed, oven cooked and fried (olive oil)	Fresh = 3.20; steamed = 8.28; oven cooked = 7.78; fried = 13.79	Fresh = 15.58; steamed = 14.64; oven cooked = 14.38; fried = 8.32	Fresh = 1.19; steamed = 1.20; oven cooked = 1.18; fried = 0.97	Fresh = 1.23; steamed = 1.23; oven cooked = 1.23; fried = 0.95	–	Fresh = 0.42; steamed = 0.42; oven cooked = 0.41; fried = 0.30	Fresh = 0.31; steamed = 0.30; oven cooked = 0.30; fried = 0.31	Fresh = 2.97; steamed = 2.99; oven cooked = 3.03; fried = 4.35	Alexi et al., 2019
	*Cynoscion leiarchus*	Braised	Fresh = 6.85; braised = 16.70	Fresh = 13.41; braised = 4.10	Fresh = 3.43; braised = 0.56	Fresh = 0.53; braised = 1.25	–	Fresh = 0.71; braised = 0.39	Fresh = 1.36; braised = 0.78	Fresh = 1.31; braised = 3.40	Brito et al., 2019
grass carp	*Ctenopharyngodon idella*	Poached, steamed, microwaved, pan fried (without oil) and deep fried (olive oil)	Fresh = 6.93; poached = 4.59; steamed = 5.81; microwaved = 6.46; pan fried = 4.81; deep fried = 19.38	Fresh = 0.77; poached = 0.67; steamed = 0.56; microwaved = 0.72; pan fried = 1.55; deep fried = 0.59	Fresh = 0.33; poached = 0.28; steamed = 0.27; microwaved = 0.26; pan fried = 0.30; deep fried = 0.35	Fresh = 0.41; poached = 0.43; steamed = 0.46; microwaved = 0.49; pan fried = 0.48; deep fried = 0.47	Fresh = 2.38; poached = 2.44; steamed = 2.39; microwaved = 2.59; pan fried = 2.79; deep fried = 2.77	Fresh = 0.52; poached = 0.53; steamed = 0.48; microwaved = 0.38; pan fried = 0.79; deep fried = 0.49	Fresh = 2.06; poached = 2.11; steamed = 2.04; microwaved = 1.87; pan fried = 1.76; deep fried = 1.91	Fresh = 1.86; poached = 1.88; steamed = 2.01; microwaved = 2.70; pan fried = 2.00; deep fried = 2.17	Golgolipour et al., 2019
yellowstripe scad	*Selaroides leptolepis*	Steamed, fried (palm oil), grilled and baked	–	Fresh = 8.06; steamed = 12.31; fried = 8.43; grilled = 11.75; baked = 12.77	–	Fresh = 0.35; steamed = 0.54; fried = 0.32; grilled = 0.48; baked = 0.52	–	–	–	–	Choo et al., 2018
Japanese threadfifin bream	*Nemipterus japonicus*	Steamed, fried (palm oil), grilled and baked	–	Fresh = 1.87; steamed = 2.90; fried = 1.26; grilled = 2.21; baked = 3.04	–	Fresh = 0.13; steamed = 0.17; fried = 0.08; grilled = 0.13; baked = 0.17	–	–	–	–	Choo et al., 2018
salmon	*Salmo salar*	Steamed, fried (palm oil), grilled and baked	–	Fresh = 2.55; steamed = 3.59; fried = 1.89; grilled = 2.89; baked = 3.51	–	Fresh = 1.01; steamed = 1.17; fried = 0.56; grilled = 1.11; baked = 1.20	–	–	–	–	Choo et al., 2018
Common carp	*Cyprinus carpio*	Smoked	Fresh = 3.41; smoked = 5.00	Fresh = 2.43; smoked = 2.58	Fresh = 0.26; smoked = 0.25	Fresh = 1.12; smoked = 1.14	Fresh = 3.53; smoked = 3.67	–	Fresh = 0.86; smoked = 0.84	Fresh = 2.92; smoked = 3.01	Ljubojevic et al., 2016
Capelin	*Mallotus villosus*	Hot-smoked and cold-smoked	Fresh = 10.2; hot-smoked = 7.4; cold-smoked = 8.2	Fresh = 12.77; hot-smoked = 17.96; cold-smoked = 17.72	–	Fresh = 0.98; hot-smoked = 1.19; cold-smoked = 1.23	Fresh = 4.00; hot-smoked = 3.85; cold-smoked = 4.18	–	Fresh = 0.64; hot-smoked = 0.54; cold-smoked = 0.52	Fresh = 1.36; hot-smoked = 1.54; cold-smoked = 1.69	Cyprian et al., 2015
Sardine	*Sardinella gibbosa*	Hot-smoked and cold-smoked	Fresh = 6.7; hot-smoked = 4.8; cold-smoked = 5.6	Fresh = 20.53; hot-smoked = 21.08; cold-smoked = 20.85	–	Fresh = 0.65; hot-smoked = 0.71; cold-smoked = 0.71	Fresh = 1.33; hot-smoked = 1.57; cold-smoked = 1.54	–	Fresh = 0.80; hot-smoked = 1.04; cold-smoked = 1.06	Fresh = 0.92; hot-smoked = 1.00; cold-smoked = 0.98	Cyprian et al., 2015
Chinook salmon	*Oncorhynchus tshawytscha*	Baked, broiled and fried	–	Fresh = 20.78; baked = 16.35; broiled = 18.55; fried = 11.29	Fresh = 2.92; baked = 2.19; broiled = 2.52; fried = 1.54	Fresh = 2.20; baked = 2.10; broiled = 2.10; fried = 2.30	–	–	–	–	Neff et al., 2014
Common carp	*Cyprinus carpio carpio*	Baked, broiled and fried	–	Fresh = 2.19; baked = 1.39; broiled = 1.07; fried = 1.62	Fresh = 0.50; baked = 0.40; broiled = 0.30; fried = 0.40	Fresh = 0.60; baked = 0.60; broiled = 0.40; fried = 0.80	–	–	–	–	Neff et al., 2014
White sucker	*Catostomus commersonii*	Baked, broiled and fried	–	Fresh = 24.31; baked = 10.95; broiled = 16.17; fried = 5.58	Fresh = 3.90; baked = 1.70; broiled = 2.30; fried = 0.90	Fresh = 1.30; baked = 1.80; broiled = 1.40; fried = 2.50	–	–	–	–	Neff et al., 2014
lake trout	*Salvelinus namaycush*	Baked, broiled and fried	–	Fresh = 15.54; baked = 13.66; broiled = 15.01; fried = 12.97	Fresh = 2.70; baked = 2.30; broiled = 2.60; fried = 2.10	Fresh = 1.60; baked = 1.60; broiled = 1.60; fried = 1.70	–	–	–	–	Neff et al., 2014
walleye	*Sander vitreus*	Baked, broiled and fried	–	Fresh = 22.41; baked = 16.65; broiled = 12.87; fried = 7.33	Fresh = 3.00; baked = 2.10; broiled = 1.80; fried = 1.00	Fresh = 1.70; baked = 1.80; broiled = 1.90; fried = 2.40	–	–	–	–	Neff et al., 2014
Kutum roach	*Rutilus frisii kutum*	Baked, boiled, microwaved and fried	Fresh = 15.49; baked = 16.95; boiled = 13.31; microwaved = 16.40; fried = 21.10	Fresh = 2.50; baked = 1.67; boiled = 1.31; microwaved = 0.96; fried = 1.19	Fresh = 3.89; baked = 1.89; boiled = 3.61; microwaved = 2.03; fried = 0.43	Fresh = 1.08; baked = 1.06; boiled = 1.03; microwaved = 1.02; fried = 1.79	Fresh = 3.63; baked = 2.93; boiled = 1.65; microwaved = 1.77; fried = 9.36	–	Fresh = 0.25; baked = 0.32; boiled = 0.21; microwaved = 0.28; fried = 0.39	Fresh = 3.09; baked = 3.13; boiled = 2.40; microwaved = 1.94; fried = 4.42	Hosseini et al., 2014
Sardine	*Sardina pilchardus*	Baked for 20–60 min	Fresh = 13.23; baked 20 min = 11.86; baked 40 min = 11.91; baked 50 min = 12.04; baked 60 min = 12.05	Fresh = 33.03; baked 20 min = 33.16; baked 40 min = 33.16; baked 50 min = 33.43; baked 60 min = 34.18	Fresh = 9.30; baked 20 min = 9.40; baked 40 min = 9.40; baked 50 min = 9.50; baked 60 min = 9.90	Fresh = 1.06; baked 20 min = 1.02; baked 40 min = 1.03; baked 50 min = 1.03; baked 60 min = 1.06	Fresh = 1.89; baked 20 min = 1.82; baked 40 min = 1.84; baked 50 min = 1.81; baked 60 min = 1.87	–	Fresh = 0.31; baked 20 min = 0.32; baked 40 min = 0.32; baked 50 min = 0.32; baked 60 min = 0.31	Fresh = 1.37; baked 20 min = 1.35; baked 40 min = 1.34; baked 50 min = 1.33; baked 60 min = 1.38	Zotos et al., 2013
Anchovy	*Engraulis encrasicholus*	Fried (olive oil) for 2 to 5 min	Fresh = 10.08; fried 2 min = 33.81; fried 3 min = 37.50; fried 4 min = 40.91; fried 5 min = 41.04	Fresh = 33.36; fried 2 min = 3.95; fried 3 min = 3.11; fried 4 min = 2.96; fried 5 min = 2.76	Fresh = 15.62; fried 2 min = 2.56; fried 3 min = 0.73; fried 4 min = 0.71; fried 5 min = 0.73	Fresh = 0.99; fried 2 min = 0.20; fried 3 min = 0.24; fried 4 min = 0.24; fried 5 min = 0.35	Fresh = 1.90; fried 2 min = 4.40; fried 3 min = 4.49; fried 4 min = 4.56; fried 5 min = 5.87	–	Fresh = 0.31; fried 2 min = 1.91; fried 3 min = 2.42; fried 4 min = 2.55; fried 5 min = 1.73	Fresh = 1.47; fried 2 min = 4.25; fried 3 min = 4.40; fried 4 min = 4.46; fried 5 min = 5.73	Zotos et al., 2013
Anchovy	*Engraulis encrasicholus*	Fried (sunflower oil) for 2 to 5 min	Fresh = 7.04; fried 2 min = 39.34; fried 3 min = 42.61; fried 4 min = 52.00; fried 5 min = 53.14	Fresh = NIL; fried 2 min = 0.21; fried 3 min = 0.25; fried 4 min = 0.24; fried 5 min = 0.24	Fresh = 0.13; fried 2 min = 35.25; fried 3 min = 22.00; fried 4 min = 26.4; fried 5 min = 27.00	Fresh = 0.53; fried 2 min = 0.11; fried 3 min = 0.11; fried 4 min = 0.11; fried 5 min = 0.11	Fresh = 2.29; fried 2 min = 9.43; fried 3 min = 10.03; fried 4 min = 11.69; fried 5 min = 11.95	–	Fresh = 0.21; fried 2 min = 0.23; fried 3 min = 0.42; fried 4 min = 0.55; fried 5 min = 0.46	Fresh = 2.06; fried 2 min = 9.27; fried 3 min = 9.97; fried 4 min = 11.63; fried 5 min = 11.89	Zotos et al., 2013
Grass carp	*Ctenopharynyodon idellus*	Boiled, steamed, microwaved, grilled, pan-fried (soybean oil) and deep-fried (soybean oil)	Fresh = 8.74; boiled = 7.46; steamed = 7.33; microwaved = 9.14; grilled = 9.65; pan-fried = 24.72; deep-fried = 25.15	Fresh = 10.73; boiled = 11.27; steamed = 10.34; microwaved = 9.95; grilled = 9.14; pan-fried = 4.34; deep-fried = 4.00	Fresh = 0.95; boiled = 0.91; steamed = 0.91; microwaved = 0.97; grilled = 0.98; pan-fried = 0.26; deep-fried = 0.25	Fresh = 2.61; boiled = 2.34; steamed = 2.52; microwaved = 2.22; grilled = 2.31; pan-fried = 3.62; deep-fried = 4.23	Fresh = 3.65; boiled = 3.35; steamed = 3.44; microwaved = 3.22; grilled = 3.27; pan-fried = 4.91; deep-fried = 5.49	Fresh = 0.22; boiled = 0.26; steamed = 0.24; microwaved = 0.27; grilled = 0.26; pan-fried = 0.19; deep-fried = 0.17	Fresh = 0.35; boiled = 0.44; steamed = 0.39; microwaved = 0.50; grilled = 0.51; pan-fried = 0.39; deep-fried = 0.34	Fresh = 3.57; boiled = 3.00; steamed = 3.30; microwaved = 2.94; grilled = 2.91; pan-fried = 4.84; deep-fried = 5.43	Zhang et al., 2013
Red mullet	*Mullus barbatus*	Oven-cooked, steamed, fried (corn oil) and microwaved	Fresh = 13.37; oven-cooked = 14.00; steamed = 13.00; fried = 34.44; microwaved = 11.00	Fresh = 1.11; oven-cooked = 0.93; steamed = 0.76; fried = 0.80; microwaved = 0.99	Fresh = 1.31; oven-cooked = 1.33; steamed = 0.03; fried = 0.64; microwaved = 1.30	Fresh = 0.08; oven-cooked = 0.06; steamed = 2.19; fried = 0.06; microwaved = 0.06	Fresh = 1.09; oven-cooked = 1.06; steamed = 4.17; fried = 0.56; microwaved = 0.84	–	Fresh = 5.35; oven-cooked = 6.89; steamed = 0.76; fried = 10.04; microwaved = 6.69	Fresh = 0.89; oven-cooked = 0.83; steamed = 4.25; fried = 0.49; microwaved = 0.69	Koubaa et al., 2012
Striped catfish	*Pangasius hypophthalmus*	Boiled, boiled with salt, microwaved with water, microwaved and fried (rapeseed oil)	Fresh = 12.14; boiled = 10.44; boiled with salt = 10.24; microwaved with water = 14.58; microwaved = 15.23; fried = 26.28	Fresh = 1.04; boiled = 1.09; boiled with salt = 0.98; microwaved with water = 1.20; microwaved = 0.95; fried = 0.29	Fresh = 0.41; boiled = 0.43; boiled with salt = 0.39; microwaved with water = 0.41; microwaved = 0.34; fried = 0.46	Fresh = 0.26; boiled = 0.47; boiled with salt = 0.26; microwaved with water = 0.25; microwaved = 0.25; fried = 1.86	Fresh = 1.39; boiled = 1.43; boiled with salt = 1.42; microwaved with water = 1.42; microwaved = 1.35; fried = 8.45	Fresh = 0.69; boiled = 0.67; boiled with salt = 0.67; microwaved with water = 0.68; microwaved = 0.71; fried = 0.12	Fresh = 2.91; boiled = 2.81; boiled with salt = 3.00; microwaved with water = 3.11; microwaved = 3.28; fried = 0.41	Fresh = 1.31; boiled = 1.35; boiled with salt = 1.36; microwaved with water = 1.34; microwaved = 1.29; fried = 8.43	Domiszewski et al., 2011
Diamon mullet	*Liza alata*	Grilled	–	Fresh = 7.69; grilled = 9.17	–	Fresh = 0.35; grilled = 0.47	Fresh = 1.15; grilled = 1.28	–	–	Fresh = 0.79; grilled = 0.90	Musaiger and D’Souza, 2011
Grey grunt	*Plectorhinchus sordidus*	Grilled	–	Fresh = 10.81; grilled = 12.69	–	Fresh = 0.69; grilled = 0.80	Fresh = 2.20; grilled = 2.10	–	–	Fresh = 1.66; grilled = 1.81	Musaiger and D’Souza, 2011
Narrow-barred Spanish mackrel	*Scomberomorus commerson*	Curried	–	Fresh = 21.33; curried = 18.96	–	Fresh = 0.78; curried = 1.13	Fresh = 1.92; curried = 2.28	–	–	Fresh = 1.41; curried = 2.27	Musaiger and D’Souza, 2011
Pear spotted rabbitfish	*Siganus canaliculatus*	Curried and fried (corn oil)	–	Fresh = 7.96; curried = 3.03; fried = 2.91	–	Fresh = 0.50; curried = 2.23; fried = 2.12	Fresh = 1.27; curried = 4.11; fried = 3.89	–	–	Fresh = 0.95; curried = 4.27; fried = 3.98	Musaiger and D’Souza, 2011
New Zealand King Salmon	*Oncorhynchus tshawytscha*	Poached, steamed, microwaved, oven baked, pan fried and deep fried	Fresh = 21.61; poached = 18.02; steamed = 21.20; microwaved = 18.32; oven baked = 24.68; pan fried = 23.14; deep fried = 26.30	Fresh = 13.06; poached = 6.03; steamed = 12.71; microwaved = 12.79; oven baked = 12.22; pan fried = 11.83; deep fried = 4.47	Fresh = 1.48; poached = 1.45; steamed = 1.42; microwaved = 1.39; oven baked = 1.37; pan fried = 1.33; deep fried = 0.56	Fresh = 1.01; poached = 1.03; steamed = 1.04; microwaved = 1.04; oven baked = 0.99; pan fried = 0.97; deep fried = 1.43	Fresh = 3.17; poached = 3.18; steamed = 3.30; microwaved = 3.20; oven baked = 3.19; pan fried = 3.18; deep fried = 3.93	–	Fresh = 0.47; poached = 0.74; steamed = 0.46; microwaved = 0.46; oven baked = 0.49; pan fried = 0.50; deep fried = 0.48	Fresh = 2.75; poached = 2.44; steamed = 2.82; microwaved = 2.74; oven baked = 2.76; pan fried = 2.72; deep fried = 3.49	Larsen et al., 2010
Indian mackerel	*Bastrilliger kanagurta*	Fried and gravy	–	Fresh = 6.42; fried = 2.99; gravy = 11.49	Fresh = 0.40; fried = 0.19; gravy = 1.01	Fresh = 0.91; fried = 0.82; gravy = 1.09	Fresh = 1.92; fried = 2.29; gravy = 1.90	Fresh = 0.50; fried = 0.42; gravy = 0.51	Fresh = 0.81; fried = 1.34; gravy = 0.57	Fresh = 1.55; fried = 1.93; gravy = 1.08	Marichamy et al., 2009
Silver catfish	*Rhamdia quelen*	Boiled, baked, microwaved, grilled, fried (soybean oil), fried (canola oil) and fried (hydrogenated vegetable oil)	Fresh = 15.50; boiled = 20.10; baked = 23.00; microwaved = 21.90; grilled = 25.70; fried (soybean oil) = 33.40; fried (canola oil) = 32.05; fried (hydrogenated vegetable oil) = 32.20	Fresh = 3.90; boiled = 4.89; baked = 3.81; microwaved = 3.76; grilled = 4.07; fried (soybean oil) = 0.73; fried (canola oil) = 1.27; fried (hydrogenated vegetable oil) = 1.37	Fresh = 0.29; boiled = 0.35; baked = 0.29; microwaved = 0.30; grilled = 0.28; fried (soybean oil) = 0.12; fried (canola oil) = 7.81; fried (hydrogenated vegetable oil) = 0.13	Fresh = 1.81; boiled = 1.77; baked = 1.86; microwaved = 1.81; grilled = 1.95; fried (soybean oil) = 4.55; fried (canola oil) = 5.39; fried (hydrogenated vegetable oil) = 1.42	Fresh = 2.38; boiled = 2.33; baked = 2.43; microwaved = 2.38; grilled = 2.55; fried (soybean oil) = 5.97; fried (canola oil) = 7.50; fried (hydrogenated vegetable oil) = 2.54	–	Fresh = 1.11; boiled = 1.03; baked = 1.10; microwaved = 1.18; grilled = 1.01; fried (soybean oil) = 0.40; fried (canola oil) = 0.42; fried (hydrogenated vegetable oil) = 2.31	Fresh = 2.27; boiled = 2.17; baked = 2.26; microwaved = 2.19; grilled = 2.42; fried (soybean oil) = 6.51; fried (canola oil) = 8.69; fried (hydrogenated vegetable oil) = 2.73	Weber et al., 2008
Atlantic hake	*Merluccius hubbsi*	Grilled	Fresh = 8.6; grilled = 7.9	Fresh = 21.89; grilled = 25.52	Fresh = 5.27; grilled = 5.19	Fresh = 1.07; grilled = 1.35	Fresh = 3.62; grilled = 3.25	Fresh = 0.23; grilled = 0.27	Fresh = 0.28; grilled = 0.33	Fresh = 3.20; grilled = 2.83	Saldanha and Bragagnolo, 2008
Indo-Pacific king mackerel	*Scomberomorous guttatus*	Microwaved, grilled, steamed and shallow fat fried (palm oil)	Fresh = 6.00; microwaved = 7.00; grilled = 10.48; steamed = 5.42; shallow fat fried = 10.30	Fresh = 18.72; microwaved = 18.51; grilled = 18.29; steamed = 18.30; shallow fat fried = 12.79	Fresh = 1.85; microwaved = 1.92; grilled = 1.82; steamed = 1.75; shallow fat fried = 0.78	Fresh = 0.89; microwaved = 0.85; grilled = 0.84; steamed = 0.87; shallow fat fried = 0.95	Fresh = 1.03; microwaved = 0.97; grilled = 0.97; steamed = 0.99; shallow fat fried = 1.10	–	Fresh = 0.35; microwaved = 0.37; grilled = 0.37; steamed = 0.37; shallow fat fried = 0.50	Fresh = 1.25; microwaved = 1.15; grilled = 1.17; steamed = 1.20; shallow fat fried = 1.28	Bakar et al., 2008
Seabass	*Dicentrarchus labrax*	Fried (sunflower oil), baked and microwaved	Fresh = 14.73; fried = 18.63; baked = 17.54; microwaved = 16.77	Fresh = 20.69; fried = 14.84; baked = 20.22; microwaved = 23.22	Fresh = 2.02; fried = 0.55; baked = 1.51; microwaved = 2.24	Fresh = 0.96; fried = 1.62; baked = 1.08; microwaved = 1.11	Fresh = 2.30; fried = 3.32; baked = 2.42; microwaved = 2.39	Fresh = 0.35; fried = 0.25; baked = 0.33; microwaved = 0.33	Fresh = 0.39; fried = 0.40; baked = 0.38; microwaved = 0.32	Fresh = 1.33; fried = 2.35; baked = 1.56; microwaved = 1.62	Türkkan et al., 2008
Rainbow trout	*Oncorhynchus mykiss*	Oven cooked and microwaved	Fresh = 8.02; oven cooked = 14.82; microwaved = 19.16	Fresh = 11.32; oven cooked = 11.59; microwaved = 11.90	Fresh = 1.94; oven cooked = 1.77; microwaved = 1.86	Fresh = 0.68; oven cooked = 0.72; microwaved = 0.76	Fresh = 1.89; oven cooked = 1.90; microwaved = 2.10	Fresh = 0.53; oven cooked = 0.48; microwaved = 0.47	Fresh = 0.65; oven cooked = 0.63; microwaved = 0.57	Fresh = 1.54; oven cooked = 1.72; microwaved = 1.81	Unusan, 2007
Norwegian trout	*Salmo trutta*	Boiled and fried (Sunflower oil)	–	Fresh = 22.94; boiled = 26.23; fried = 26.82	Fresh = 4.81; boiled = 4.26; fried = 5.24	Fresh = 1.35; boiled = 1.45; fried = 1.73	Fresh = 3.20; boiled = 3.64; fried = 2.01	–	Fresh = 0.16; boiled = 0.15; fried = 0.11	Fresh = 2.46; boiled = 3.08; fried = 2.83	Gladyshev et al., 2007
Siberian trout	*Salmo trutta*	Boiled and fried (Sunflower oil)	–	Fresh = 30.98; boiled = 48.44; fried = 35.18	Fresh = 7.82; boiled = 5.88; fried = 4.86	Fresh = 1.57; boiled = 1.62; fried = 2.00	Fresh = 4.41; boiled = 3.81; fried = 2.24	–	Fresh = 0.10; boiled = 0.12; fried = 0.10	Fresh = 2.24; boiled = 2.67; fried = 2.94	Gladyshev et al., 2007
Herring	*Clupea harengus pallasi*	Boiled and fried (Sunflower oil)	–	Fresh = 30.00; boiled = 23.98; fried = 30.99	Fresh = 19.70; boiled = 21.10; fried = 9.39	Fresh = 1.01; boiled = 0.67; fried = 1.09	Fresh = 3.25; boiled = 2.62; fried = 1.94	–	Fresh = 0.06; boiled = 0.07; fried = 0.08	Fresh = 2.16; boiled = 1.57; fried = 2.15	Gladyshev et al., 2007
Sole	*Lepidopsetta bilineata*	Boiled and fried (Sunflower oil)	–	Fresh = 33.09; boiled = 33.40; fried = 31.88	Fresh = 9.97; boiled = 7.92; fried = 1.87	Fresh = 1.05; boiled = 0.81; fried = 1.06	Fresh = 3.39; boiled = 4.24; fried = 3.01	–	Fresh = 0.07; boiled = 0.10; fried = 0.22	Fresh = 2.48; boiled = 2.76; fried = 2.96	Gladyshev et al., 2007
Cod	*Gadus morhua marisalbi*	Boiled and fried (Sunflower oil)	–	Fresh = 51.69; boiled = 53.16	Fresh = 43.70; boiled = 41.30	Fresh = 1.42; boiled = 1.74	Fresh = 2.16; boiled = 2.84	–	Fresh = 0.01; boiled = 0.01	Fresh = 2.77; boiled = 3.45	Gladyshev et al., 2007
Common carp	*Cyprinus carpio*	Roasted and steamed	–	Fresh = 1.5; roasted = 0.6; steamed = 1.5	Fresh = 0.18; roasted = 0.10; steamed = 0.17	Fresh = 0.60; roasted = 0.50; steamed = 0.60	Fresh = 3.47; roasted = 3.12; steamed = 3.27		Fresh = 1.96; roasted = 2.71; steamed = 2.09	Fresh = 2.96; roasted = 2.41; steamed = 2.84	de Castro et al., 2007
Nile tilapia	*Oreochromis niloticus*	Roasted and steamed	–	Fresh = 3.10; roasted = 3.40; steamed = 3.70	Fresh = 0.73; roasted = 0.71; steamed = 0.80	Fresh = 0.35; roasted = 0.36; steamed = 0.37	Fresh = 1.59; roasted = 1.43; steamed = 1.60	Fresh = 0.62; roasted = 0.67; steamed = 0.61	Fresh = 1.98; roasted = 1.88; steamed = 1.79	Fresh = 1.17; roasted = 1.01; steamed = 1.20	de Castro et al., 2007
Tambacu	*Colossoma macropomum* × *Piaractus mesopotamicus*	Roasted and steamed	–	Fresh = 3.00; roasted = 3.20; steamed = 2.90	Fresh = 0.59; roasted = 0.60; steamed = 0.74	Fresh = 0.40; roasted = 0.40; steamed = 0.40	Fresh = 1.65; roasted = 1.45; steamed = 1.33	–	Fresh = 1.73; roasted = 1.70; steamed = 1.49	Fresh = 1.43; roasted = 1.23; steamed = 1.04	de Castro et al., 2007
Cod	*Gadus morhua*	Fried (margarine), fried (olive oil)	–	Fresh = 49.61; fried (margarine) = 9.83; fried (olive oil) = 12.27	Fresh = 10.00; fried (margarine) = 1.37; fried (olive oil) = 2.50	Fresh = 2.16; fried (margarine) = 0.33; fried (olive oil) = 1.16	Fresh = 3.34; fried (margarine) = 1.04; fried (olive oil) = 9.88	Fresh = 0.27; fried (margarine) = 0.84; fried (olive oil) = 0.18	Fresh = 0.14; fried (margarine) = 0.91; fried (olive oil) = 0.35	Fresh = 3.45; fried (margarine) = 1.55; fried (olive oil) = 5.24	Sioen et al., 2006
Salmon	*Salmo salar*	Fried (margarine), fried (olive oil)	–	Fresh = 19.17; fried (margarine) = 17.96; fried (olive oil) = 15.21	Fresh = 3.33; fried (margarine) = 3.13; fried (olive oil) = 3.13	Fresh = 1.30; fried (margarine) = 1.03; fried (olive oil) = 1.14	Fresh = 3.80; fried (margarine) = 3.05; fried (olive oil) = 4.20	Fresh = 0.26; fried (margarine) = 0.15; fried (olive oil) = 0.23	Fresh = 0.31; fried (margarine) = 0.19; fried (olive oil) = 0.36	Fresh = 2.17; fried (margarine) = 4.63; fried (olive oil) = 2.38	Sioen et al., 2006
humpback salmon	*Oncorhynchus gorbuscha*	Boiled, fried (sunflower oil), and roasted	–	Fresh = 26.45; boiled = 28.40; fried = 20.58; roasted = 19.64	Fresh = 16.2; boiled = 15.9; fried=;2.2 roasted = 3.4	–	Fresh = 4.36; boiled = 5.31; fried = 4.02; roasted = 7.41	–	Fresh = 0.11; boiled = 0.13; fried = 0.31; roasted = 0.43	Fresh = 2.24; boiled = 2.41; fried = 3.15; roasted = 2.10	Gladyshev et al., 2006
Sardine	*Sardina pilchardus*	Fried (olive oil), baked and grilled	Fresh = 39.25; fried = 37.33; baked = 32.99; grilled = 33.35	Fresh = 29.45; fried = 12.71; baked = 30.22; grilled = 29.76	Fresh = 26.76; fried = 3.83; baked = 26.35; grilled = 27.73	Fresh = 1.16; fried = 0.95; baked = 1.14; grilled = 1.25	Fresh = 2.36; fried = 4.46; baked = 2.35; grilled = 2.53	–	Fresh = 0.10; fried = 0.31; baked = 0.10; grilled = 0.10	Fresh = 1.87; fried = 4.31; baked = 1.90; grilled = 1.99	Garcia-Arias et al., 2003
Herring		Boiled (microwave), boiled (conventionally), grilled (microwave), grilled (conventioanally), fried (microwave + “Smazyk” cooking oil) and fried (conventionally + “Smazyk” cooking oil)	–	Fresh = 13.80; boiled (microwave) = 15.00; boiled (conventionally) = 12.90; grilled (microwave) = 12.70; grilled (conventioanally) = 14.50; fried (microwave + “Smazyk” cooking oil) = 13.60; fried (conventionally + “Smazyk” cooking oil) = 13.70	–	Fresh = 0.93; boiled (microwave) = 0.94; boiled (conventionally) = 0.91; grilled (microwave) = 0.91; grilled (conventioanally) = 1.01; fried (microwave + “Smazyk” cooking oil) = 0.85; fried (conventionally + “Smazyk” cooking oil) = 1.16	Fresh = 2.13; boiled (microwave) = 2.03; boiled (conventionally) = 2.21; grilled (microwave) = 2.17; grilled (conventioanally) = 2.33; fried (microwave + “Smazyk” cooking oil) = 1.90; fried (conventionally + “Smazyk” cooking oil) = 2.49	–	–	Fresh = 1.47; boiled (microwave) = 1.43; boiled (conventionally) = 1.53; grilled (microwave) = 1.51; grilled (conventioanally) = 1.59; fried (microwave + “Smazyk” cooking oil) = 1.34; fried (conventionally + “Smazyk” cooking oil) = 1.70	Regulska-Ilow and Ilow, 2002
Salmon		Fried (olive oil), fried (soya oil) and roasted	Fresh = 9.76; fried (olive oil) = 19.54; fried (soya oil) = 17.97; roasted = 8.57	Fresh = 17.51; fried (olive oil) = 16.73; fried (soya oil) = 15.94; roasted = 15.85	Fresh = 6.48; fried (olive oil) = 4.27; fried (soya oil) = 3.23; roasted = 3.34	Fresh = 1.29; fried (olive oil) = 1.21; fried (soya oil) = 1.39; roasted = 1.57	Fresh = 2.76; fried (olive oil) = 2.47; fried (soya oil) = 2.60; roasted = 2.77	–	–	Fresh = 2.12; fried (olive oil) = 2.11; fried (soya oil) = 2.23; roasted = 2.69	Echarte et al., 2001
	*Salmon salar*	Fried (sunflower oil) and warmheld	–	Fresh = 18.83; fried = 15.24; warmheld = 17.74	Fresh = 8.33; fried = 0.93; warmheld = 1.61	Fresh = 0.78; fried = 1.45; warmheld = 1.24	Fresh = 2.49; fried = 3.56; warmheld = 3.23	–	Fresh = 0.39; fried = 0.39; warmheld = 0.37	Fresh = 1.71; fried = 2.86; warmheld = 2.46	Candela et al., 1998
Spanish mackerel	*Scomberomorus commersoni*	Fried (sunflower oil) and warmheld	–	Fresh = 24.98; fried = 6.91; warmheld = 5.43	Fresh = 8.33; fried = 0.16; warmheld = 0.13	Fresh = 0.98; fried = 3.55; warmheld = 3.49	Fresh = 2.42; fried = 7.02; warmheld = 7.08	–	Fresh = 0.32; fried = 0.32; warmheld = 0.35	Fresh = 1.62; fried = 7.15; warmheld = 7.26	Candela et al., 1998
sardine	*Sardine pilchardus*	Fried (sunflower oil) and warmheld	–	Fresh = 31.81; fried = 7.88; warmheld = 7.80	Fresh = 14.29; fried = 0.17; warmheld = 0.17	Fresh = 1.04; fried = 3.62; warmheld = 3.58	Fresh = 2.00; fried = 6.76; warmheld = 6.65	–	Fresh = 0.30; fried = 0.31; warmheld = 0.31	Fresh = 1.54; fried = 7.01; warmheld = 6.90	Candela et al., 1998
sardine	*Sardine pilchardus*	Fried (olive oil)	Fresh = 3.93; fried = 3.73	Fresh = 29.36; fried = 12.77	Fresh = 4.05; fried = 2.08	Fresh = 1.34; fried = 1.09	Fresh = 2.21; fried = 4.34	–	Fresh = 0.25; fried = 0.37	Fresh = 2.16; fried = 4.51	Castrillion et al., 1997
Rainbow trout	*Oncorhynchus mykiss*	Boiled, baked, microwaved, fried (sunflower oil) and fried (rapeseed oil)	Fresh = 10.55; boiled = 15.05; baked = 12.69; microwaved = 15.09; fried (sunflower oil) = 14.61; fried (rapeseed oil) = 19.63	Fresh = 28.81; boiled = 23.79; baked = 25.24; microwaved = 24.05; fried (sunflower oil) = 18.48; fried (rapeseed oil) = 20.76	Fresh = 6.45; boiled = 4.98; baked = 5.18; microwaved = 5.03; fried (sunflower oil) = 1.14; fried (rapeseed oil) = 3.63	Fresh = 2.05; boiled = 1.78; baked = 1.70; microwaved = 36.89; fried (sunflower oil) = 2.52; fried (rapeseed oil) = 1.62	Fresh = 4.62; boiled = 4.39; baked = 3.74; microwaved = 3.73; fried (sunflower oil) = 5.55; fried (rapeseed oil) = 4.22	–	Fresh = 0.18; boiled = 0.22; baked = 0.23; microwaved = 0.25; fried (sunflower oil) = 0.22; fried (rapeseed oil) = 0.25	Fresh = 3.32; boiled = 3.03; baked = 2.83; microwaved = 2.6; fried (sunflower oil) = 4.34; fried (rapeseed oil) = 3.01	Agren and Hamminen, 1993
Vendace	*Coregonus albula*	Boiled, baked, microwaved, fried (sunflower oil) and fried (rapeseed oil)	Fresh = 3.52; boiled = 5.17; baked = 4.85; microwaved = 6.04; fried (sunflower oil) = 23.94; fried (rapeseed oil) = 23.46	Fresh = 38.81; boiled = 25.74; baked = 32.12; microwaved = 30.14; fried (sunflower oil) = 5.38; fried (rapeseed oil) = 6.41	Fresh = 3.83; boiled = 3.53; baked = 3.51; microwaved = 3.38; fried (sunflower oil) = 0.15; fried (rapeseed oil) = 0.70	Fresh = 2.39; boiled = 2.25; baked = 1.26; microwaved = 2.24; fried (sunflower oil) = 4.69; fried (rapeseed oil) = 3.95	Fresh = 3.71; boiled = 3.55; baked = 3.65; microwaved = 3.72; fried (sunflower oil) = 9.76; fried (rapeseed oil) = 12.90	–	Fresh = 0.17; boiled = 0.18; baked = 0.14; microwaved = 0.14; fried (sunflower oil) = 0.44; fried (rapeseed oil) = 0.15	Fresh = 3.13; boiled = 2.76; baked = 3.20; microwaved = 2.91; fried (sunflower oil) = 9.32; fried (rapeseed oil) = 11.96	Agren and Hamminen, 1993
Pike	*Esox lucius*	Boiled, baked, microwaved, fried (sunflower oil) and fried (rapeseed oil)	Fresh = 1.93; boiled = 2.19; baked = 1.56; microwaved = 1.43; fried (sunflower oil) = 7.43; fried (rapeseed oil) = 9.89	Fresh = 57.21; boiled = 55.13; baked = 51.74; microwaved = 57.21; fried (sunflower oil) = 15.52; fried (rapeseed oil) = 6.07	Fresh = 11.18; boiled = 10.38; baked = 7.80; microwaved = 8.74; fried (sunflower oil) = 0.34; fried (rapeseed oil) = 0.69	Fresh = 2.73; boiled = 2.62; baked = 0.72; microwaved = 2.99; fried (sunflower oil) = 4.25; fried (rapeseed oil) = 3.69	Fresh = 4.04; boiled = 3.87; baked = 4.08; microwaved = 4.49; fried (sunflower oil) = 7.67; fried (rapeseed oil) = 15.05	–	Fresh = 0.13; boiled = 0.14; baked = 0.14; microwaved = 0.12; fried (sunflower oil) = 0.21; fried (rapeseed oil) = 0.16	Fresh = 3.94; boiled = 3.75; baked = 3.98; microwaved = 4.37; fried (sunflower oil) = 7.62; fried (rapeseed oil) = 14.37	Agren and Hamminen, 1993
grouper	*Epinephelus morio*	Baked, broiled, deep fried (soybean oil) and microwaved	Fresh = 0.88; baked = 1.14; broiled = 1.18; deep fried = 3.73; microwaved = 1.41	Fresh = 27.06; baked = 28.00; broiled = 26.34; deep fried = 6.06; microwaved = 16.71	–	Fresh = 1.35; baked = 1.30; broiled = 1.43; deep fried = 2.36; microwaved = 1.06	Fresh = 2.56; baked = 2.45; broiled = 2.47; deep fried = 4.01; microwaved = 2.08	–	Fresh = 0.30; baked = 0.29; broiled = 0.28; deep fried = 0.33; microwaved = 0.41	Fresh = 2.31; baked = 2.25; broiled = 2.33; deep fried = 4.50; microwaved = 1.99	Gall et al., 1983
red snapper	*Lutjanus campechanus*	Baked, broiled, deep fried (soybean oil) and microwaved	Fresh = 1.50; baked = 1.31; broiled = 1.75; deep fried = 5.49; microwaved = 1.65	Fresh = 26.41; baked = 28.33; broiled = 27.01; deep fried = 7.53; microwaved = 29.79	–	Fresh = 1.27; baked = 1.39; broiled = 1.42; deep fried = 2.60; microwaved = 1.34	Fresh = 2.36; baked = 2.40; broiled = 2.49; deep fried = 2.89; microwaved = 4.13	–	Fresh = 0.28; baked = 0.25; broiled = 0.26; deep fried = 0.27; microwaved = 0.27	Fresh = 2.34; baked = 2.58; broiled = 2.50; deep fried = 5.58; microwaved = 2.42	Gall et al., 1983
Florida pompano	*Trachinotus carolinus*	Baked, broiled, deep fried (soybean oil) and microwaved	Fresh = 5.17; baked = 4.48; broiled = 4.19; deep fried = 8.78; microwaved = 4.21	Fresh = 9.32; baked = 9.32; broiled = 11.14; deep fried = 4.81; microwaved = 9.61	–	Fresh = 0.56; baked = 0.56; broiled = 0.61; deep fried = 1.47; microwaved = 0.61	Fresh = 1.58; baked = 1.55; broiled = 1.57; deep fried = 1.86; microwaved = 2.65	–	Fresh = 0.77; baked = 0.78; broiled = 0.70; deep fried = 0.51; microwaved = 0.73	Fresh = 1.00; baked = 0.97; broiled = 1.02; deep fried = 2.39; microwaved = 1.06	Gall et al., 1983
Spanish mackerel	*Scomberomorus macuhtus*	Baked, broiled, deep fried (soybean oil) and microwaved	Fresh = 13.75; baked = 12.65; broiled = 13.18; deep fried = 12.42; microwaved = 13.61	Fresh = 21.14; baked = 19.31; broiled = 21.42; deep fried = 19.34; microwaved = 19.63	–	Fresh = 0.99; baked = 0.83; broiled = 0.88; deep fried = 1.29; microwaved = 0.85	Fresh = 2.11; baked = 2.08; broiled = 2.13; deep fried = 2.32; microwaved = 2.61	–	Fresh = 0.43; baked = 0.44; broiled = 0.40; deep fried = 0.40; microwaved = 0.43	Fresh = 2.03; baked = 2.02; broiled = 2.06; deep fried = 2.83; microwaved = 2.03	Gall et al., 1983

**Table 2:** Effects of culinary treatments on the lipid nutritional quality of shellfish.

**Common name**	**Species**	**Cooking methods**	**Total lipid (% DW)**	**EPA + DHA (%)**	**n3/n6**	**PUFA/SFA**	**(MUFA + PUFA)/(SFA - C18:0)**	**AI**	**TI**	**H/H**	**References**
Green tiger shrimp	*Penaeus semisulcatus*	Salted, fried, grilled and boiled	Fresh = 14.30; salted = 12.28; fried = 19.92; grilled = 14.90; boiled = 15.33	–	Fresh = 2.44; salted = 2.38; fried = 1.64; grilled = 2.38; boiled = 2.50	Fresh = 1.45; salted = 1.45; fried = 1.63; grilled = 1.42; boiled = 1.44	–	–	–	–	AlFaris et al., 2022
Mediterranean mussel	*Mytilus galloprovincialis*	Grilled, boiled, microwaved, oven cooked and fried (sunflower oil)	Fresh = 9.50; grilled = 10.00; boiled = 9.50; microwaved = 8.00; oven cooked = 10.00; fried = 23.00	Fresh = 24.06; grilled = 20.51; boiled = 21.20; microwaved = 20.11; oven cooked = 18.32; fried = 4.60	Row = 6.01; grilled = 3.23; boiled = 3.84; microwaved = 3.64; oven cooked = 3.43; fried = 0.15	Fresh = 0.92; grilled = 1.14; boiled = 1.02; microwaved = 0.97; oven cooked = 1.00; fried = 35.37	Fresh = 1.38; grilled = 2.12; boiled = 1.83; microwaved = 1.77; oven cooked = 1.95; fried = 6.68	Fresh = 1.09; grilled = 0.69; boiled = 0.78; microwaved = 0.78; oven cooked = 0.74; fried = 0.19	Fresh = 0.34; grilled = 0.31; boiled = 0.33; microwaved = 0.34; oven cooked = 0.34; fried = 0.23	Fresh = 1.04; grilled = 1.39; boiled = 1.20; microwaved = 1.31; oven cooked = 1.29; fried = 6.33	Biandolino et al., 2021
Pacific oyster	*Crassostrea gigas*	Fried (olive oil), gratin	Fresh = 13.7; fried = 14.19; gratin = 14.8	Fresh = 38.92; fried = 32.54; gratin = 32.49	Fresh = 5.33; fried = 3.58; gratin = 3.29	Fresh = 1.83; fried = 1.84; gratin = 1.75	Fresh = 2.60; fried = 3.21; gratin = 3.07	–	Fresh = 0.18; fried = 0.20; gratin = 0.22	Fresh = 2.58; fried = 3.06; gratin = 3.07	Felici et al., 2019
Carpet shell	*Venerupis decussata*	Fried (corn oil), fried (olive oil) and fried (margarine)	–	Fresh = 17.41; fried (corn oil) = 10.27; fried (olive oil) = 12.02; fried (margarine) = 4.06	Fresh = 5.30; fried (corn oil) = 1.29; fried (olive oil) = 1.62; fried (margarine) = 0.56	Fresh = 0.85; fried (corn oil) = 0.77; fried (olive oil) = 0.88; fried (margarine) = 0.55	Fresh = 1.67; fried (corn oil) = 1.48; fried (olive oil) = 1.98; fried (margarine) = 0.01	–	Fresh = 0.47; fried (corn oil) = 0.80; fried (olive oil) = 0.62; fried (margarine) = 1.52	Fresh = 1.12; fried (corn oil) = 0.99; fried (olive oil) = 1.37; fried (margarine) = 0.83	Bejaoui et al., 2019
Rapa whelk	*Rapana venosa*	Boiled	Fresh = 5.10; boiled = 6.22	Fresh = 19.95; boiled = 17.81	Fresh = 1.43; boiled = 1.35	Fresh = 0.95; boiled = 0.93	Fresh = 1.59; boiled = 1.59	Fresh = 0.81; boiled = 0.79	Fresh = 0.44; boiled = 0.46	Fresh = 0.97; boiled = 0.92	Merdzhanova et al., 2018
Clam	mix	Steamed and fried	–	Fresh = 11.35; steamed = 14.56; fried = 1.21	–	Fresh = 1.00; steamed = 2.89; fried = 1.07	–	–	–	–	Wright et al., 2018
Mediterranean mussel	*Mytilus galloprovincialis*	Steamed	–	Fresh = 19.69; steamed = 17.46	–	Fresh = 1.42; steamed = 1.42	–	–	–	–	Wright et al., 2018
Oyster	estern oyster	Steamed and oven dried	–	Fresh = 18.30; steamed = 18.25; oven dried = 18.26	–	Fresh = 1.13; steamed = 1.12; oven dried = 1.12	–	–	–	–	Wright et al., 2018
Pacific oyster	*Crassostrea gigas*	Steamed	–	Fresh = 29.48; steamed = 29.91	–	Fresh = 1.75; steamed = 1.75	–	–	–	–	Wright et al., 2018
Scallop	mix	Steamed and fried	–	Fresh = 21.02; steamed = 20.95; fried = 1.73	–	Fresh = 1.00; steamed = 1.00; fried = 1.07	–	–	–	–	Wright et al., 2018
Noah's ark shell	*Arca noae*	Steamed, boiled, grilled and fried (olive oil)	–	Fresh = 8.02; steamed = 8.03; boiled = 9.03; grilled = 7.33; fried = 3.05	Fresh = 2.66; steamed = 1.46; boiled = 1.85; grilled = 1.38; fried = 0.27	Fresh = 1.03; steamed = 1.29; boiled = 0.90; grilled = 1.07; fried = 0.75	Fresh = 1.56; steamed = 1.99; boiled = 1.48; grilled = 1.67; fried = 3.50	Fresh = 1.18; steamed = 1.01; boiled = 1.22; grilled = 1.13; fried = 0.42	Fresh = 0.42; steamed = 0.45; boiled = 0.51; grilled = 0.54; fried = 0.54	Fresh = 1.07; steamed = 1.05; boiled = 0.88; grilled = 1.06; fried = 3.42	Ghribi et al., 2017
Blue mussel	*Mytilus edulis*	Fried (sunflower oil)	Fresh = 0.72; fried = 32.42	Fresh = 27.27; fried = 2.96	Fresh = 3.52; fried = 0.07	Fresh = 0.94; fried = 5.42	Fresh = 2.08; fried = 2.52	–	Fresh = 0.42; fried = 0.28	Fresh = 1.51; fried = 11.09	Czech et al., 2015
Fresh water clam	*Corbicula javanica*	Boiled, steamed and boiled with salt	Fresh = 4.99; boiled = 2.83; steamed = 3.09 and boiled with salt = 1.98	Fresh = 3.82; boiled = 2.39; steamed = 2.91; boiled with salt = 2.19	Fresh = 1.48; boiled = 2.14; steamed = 1.71; boiled with salt = 1.85	Fresh = 0.44; boiled = 0.36; steamed = 0.40; boiled with salt = 0.37	Fresh = 1.52; boiled = 1.64; steamed = 1.61; boiled with salt = 1.82	Fresh = 0.65; boiled = 0.70; steamed = 0.64; boiled with salt = 0.66	Fresh = 0.66; boiled = 0.58; steamed = 0.62; boiled with salt = 0.63	Fresh = 1.13; boiled = 0.98; steamed = 1.08; boiled with salt = 0.78	Purwaningsih et al., 2015
Obtuse horn shell	*Cerithide obtusa*	Boiled, steamed and boiled with salt	Fresh = 4.71; boiled = 1.81; steamed = 2.26; boiled with salt = 1.76	Fresh = 5.34; boiled = 2.71; steamed = 3.16; boiled with salt = 1.81	Fresh = 0.84; boiled = 0.68; steamed = 0.71; boiled with salt = 0.67	Fresh = 1.81; boiled = 1.72; steamed = 2.03; boiled with salt = 1.17	Fresh = 12.71; boiled = 13.54; steamed = 17.62; boiled with salt = 8.57	Fresh = 0.07; boiled = 0.03; steamed = 0.03; boiled with salt = 0.05	Fresh = 0.17; boiled = 0.17; steamed = 0.16; boiled with salt = 0.25	Fresh = 14.72; boiled = 30.75; steamed = 32.12; boiled with salt = 19.32	Purwaningsih et al., 2015
Golden apple snail	*Pomacea canaliculata*	Boiled, steamed and boiled with salt	Fresh = 4.38; boiled = 1.24; steamed = 2.70; boiled with salt = 1.24	Fresh = 8.00; boiled = 5.84; steamed = 7.75; boiled with salt = 5.13	Fresh = 0.82; boiled = 0.68; steamed = 0.85; boiled with salt = 0.70	Fresh = 0.78; boiled = 0.81; steamed = 0.80; boiled with salt = 0.92	Fresh = 2.60; boiled = 2.33; steamed = 2.36; boiled with salt = 2.50	Fresh = 0.32; boiled = 0.36; steamed = 0.32; boiled with salt = 0.33	Fresh = 0.43; boiled = 0.47; steamed = 0.37; boiled with salt = 0.41	Fresh = 2.94; boiled = 2.55; steamed = 2.98; boiled with salt = 2.77	Purwaningsih et al., 2015
white shrimp	*Penaeus setiferus*	Fried (sunflower oil)	Fresh = 0.40; fried = 39.78	Fresh = 23.02; fried = 2.73	Fresh = 1.49; fried = 0.05	Fresh = 1.11; fried = 6.13	Fresh = 2.72; fried = 11.06	–	Fresh = 0.44; fried = 0.26	Fresh = 2.25; fried = 10.96	Czech et al., 2015
Disk abalone	*Haliotis discus*	Heating in water bath at 60–100 °C for 2 h	Fresh=; 60 °C=; 70 °C=; 80 °C=; 90 °C=; 100 °C=	Fresh = 14.74; 60 °C = 15.67; 70 °C = 17.22; 80 °C = 16.32; 90 °C = 16.00; 100 °C = 15.88	Fresh = 0.89; 60 °C = 1.03; 70 °C = 1.03; 80 °C = 1.00; 90 °C = 1.00; 100 °C = 0.94	Fresh = 0.76; 60 °C = 0.83; 70 °C = 0.87; 80 °C = 0.81; 90 °C = 0.77; 100 °C = 0.82	Fresh = 1.74; 60 °C = 1.82; 70 °C = 1.87; 80 °C = 1.80; 90 °C = 1.70; 100 °C = 1.81	–	Fresh = 0.73; 60 °C = 0.63; 70 °C = 0.60; 80 °C = 0.66; 90 °C = 0.69; 100 °C = 0.66	Fresh = 1.20; 60 °C = 1.32; 70 °C = 1.35; 80 °C = 1.28; 90 °C = 1.22; 100 °C = 1.28	Wang et al., 2014
Australian scallop	*Pecten fumatus*	Steamed, deep-fried in batter and pan fried (vegetable oil)	–	Fresh = 19.94; steamed = 23.39; deep-fried in batter = 14.46; pan fried (vegetable oil) = 18.06	Fresh = 6.81; steamed = 7.04; deep-fried in batter = 3.35; pan fried (vegetable oil) = 5.36	Fresh = 1.31; steamed = 1.46; deep-fried in batter = 1.56; pan fried (vegetable oil) = 1.48	–	–	–	–	Su and Babb, 2007
Mediterranean mussel	*Mytilus galloprovincialis*	Fresh, steamed, fried (sunflower oil), pickled and smoked	–	Fresh = 26.40; steamed = 13.20; fried = 1.50; pickled = 21.8; smoked = 16.7	–	Fresh = 1.36; steamed = 3.23; fried = 2.20; pickled = 0.96; smoked = 1.27	Fresh = 3.29; steamed = 6.08; fried = 4.05; pickled = 2.28; smoked = 2.07	–	–	–	Otles and Sengor, 2005

## Conclusion

In a nutshell, high temperature and long-term heat treatment during culinary preparation reduced EPA + DHA and n-3/n-6 of fish and shellfish, but might increase PUFA/SFA and (MUFA + PUFA)/SFA-C18:0 through induced hydrolysis of pro-atherogenic SFA. Frying has the most dynamic impact on the lipid nutritional quality of fish and shellfish, which largely depends on the frying medium used, mainly attributed to exchange of fatty acids between cooking oil and seafood. Among culinary treatments, braising, curry cooking, graving, canola oil frying, rapeseed oil frying and soybean oil frying are highly recommended in cooking fish, whereas microwave cooking, oven cooking, olive oil frying and sunflower oil frying are recommended for cooking shellfish. The worse culinary treatment for fish and shellfish is frying using margarine.

## Declaration of Competing Interest

The authors declare that they have no known competing financial interests or personal relationships that could have appeared to influence the work reported in this paper.

## Data Availability

Data will be made available on request.
