# Supplementary material for: Effects of culinary treatments on the lipid nutritional quality of fish and shellfish
Source: Food Chem X. 2023 Sep 4;19:100856. doi: 10.1016/j.fochx.2023.100856 (PMC10534239; doi:10.1016/j.fochx.2023.100856)
Supplement: Supplementary data 1 [file mmc1.docx]

Table S1: Recommendation of culinary treatments for fish and shellfish

|  | EPA+DHA | n-3/n-6 | PUFA/SFA | (MUFA+PUFA)/SFA-C18:0 | AI | TI | HH |
| --- | --- | --- | --- | --- | --- | --- | --- |
| **Recommended culinary treatments for fish** |  |  |  |  |  |  |  |
| Braising | XXX | XXX | √√ | - | √√√ | √√√ | √√√ |
| Curry cooking | XX | - | √√ | √√ | - | - | √√√ |
| Graving | √ | √ | - | - | - | √ | X |
| Frying with canola oil | XX | √√√ | √ | √ | - | √√ | √√√ |
| Frying with rapeseed oil | XX | - | √ | √ | √√√ | √√ | √√ |
| Frying with soybean oil | XX | XX | √ | √ | - | √ | √ |
|  |  |  |  |  |  |  |  |
| **Not recommended culinary treatments for fish** |  |  |  |  |  |  |  |
| Frying with margarine | XXX | XXX | XXX | XXX | XX | XXX | XXX |
|  |  |  |  |  |  |  |  |
|  |  |  |  |  |  |  |  |
| **Recommended culinary treatments for shellfish** |  |  |  |  |  |  |  |
| Microwave cooking | - | X | - | - | √ | - | √√ |
| Oven cooking | - | X | - | - | √ | - | √√ |
| Frying with olive oil | X | XX | - | - | √ | X | √ |
| Frying with sunflower oil | XXX | XXX | √ | √ | √√√ | √√√ | √√√ |
|  |  |  |  |  |  |  |  |
| **Not recommended culinary treatments for shellfish** |  |  |  |  |  |  |  |
| Frying with margarine | XX | XX | XXX | XXX | NIL | XXX | XXX |
| Frying with corn oil | X | X | X | - | NIL | XX | X |

“-” indicates no significant different; “NIL” indicates no report; “√” indicates increased slightly with significantly difference; “√√” indicates increased moderately with significant difference; “√√√” indicates increased dramatically with significantly difference; “X” indicates decreased slightly with significantly difference; “XX” indicates decreased moderately with significant difference; “XXX” indicates decreased dramatically with significantly difference.
